# Gene identification and transcriptome analysis of low cadmium accumulation rice mutant (*lcd1*) in response to cadmium stress using MutMap and RNA-seq

**DOI:** 10.1186/s12870-019-1867-y

**Published:** 2019-06-11

**Authors:** Zhen Zhen Cao, Xiao Yan Lin, Yong Jie Yang, Mei Yan Guan, Ping Xu, Ming Xue Chen

**Affiliations:** Rice Product Quality Supervision and Inspection Center, China National Rice Research Institute, Hangzhou 310006, PR, No.28 Shuidaosuo Rd., Fuyang, 311400 Zhejiang China

**Keywords:** Rice (*Oryza sativa* L.), Cadmium, MutMap, RNA-seq, SNP, Transcriptome analysis

## Abstract

**Background:**

Cadmium (Cd) is a widespread toxic heavy metal pollutant in agricultural soil, and Cd accumulation in rice grains is a major intake source of Cd for Asian populations that adversely affect human health. However, the molecular mechanism underlying Cd uptake, translocation and accumulation has not been fully understood in rice plants.

**Results:**

In this study, a mutant displaying extremely low Cd accumulation (*lcd1*) in rice plant and grain was generated by EMS mutagenesis from *indica* rice cultivar 9311 seeds. The candidate SNPs associated with low Cd accumulation phenotype in the *lcd1* mutant were identified by MutMap and the transcriptome changes between *lcd1* and WT under Cd exposure were analyzed by RNA-seq. The *lcd1* mutant had lower Cd uptake and accumulation in rice root and shoot, as well as less growth inhibition compared with WT in the presence of 5 μM Cd. Genetic analysis showed that *lcd1* was a single locus recessive mutation. The SNP responsible for low Cd accumulation in the *lcd1* mutant located at position 8,887,787 on chromosome 7, corresponding to the seventh exon of *OsNRAMP5*. This SNP led to a Pro236Leu amino acid substitution in the highly conserved region of OsNRAMP5 in the *lcd1* mutant. A total of 1208 genes were differentially expressed between *lcd1* and WT roots under Cd exposure, and DEGs were enriched in transmembrane transport process GO term. Increased *OsHMA3* expression probably adds to the effect of *OsNRAMP5* mutation to account for the significant decreases in Cd accumulation in rice plant and grain of the *lcd1* mutant.

**Conclusions:**

An extremely low Cd mutant *lcd1* was isolated and identified using MutMap and RNA-seq. A Pro236Leu amino acid substitution in the highly conserved region of OsNRAMP5 is likely responsible for low Cd accumulation in the *lcd1* mutant. This work provides more insight into the mechanism of Cd uptake and accumulation in rice, and will be helpful for developing low Cd accumulation rice by marker-assisted breeding.

**Electronic supplementary material:**

The online version of this article (10.1186/s12870-019-1867-y) contains supplementary material, which is available to authorized users.

## Background

Cadmium (Cd) is a widespread toxic soil pollutant, which is easily taken up by crop plants and accumulates in the edible parts, thus posing potential health risks for humans [1]. Rice (*Oryza sativa* L.), as staple food for half of the world’s population, is a major source of Cd intake for Chinese and Japanese populations, accounting for 40–50% of the daily Cd intake [2–4]. Thus, it is urgent to develop strategies to reduce rice grain Cd accumulation and the associated health risk of Cd intake. Selection and breeding of low Cd-accumulation rice varieties is one of the most economical and effective approaches to alleviating Cd toxicity and minimize Cd accumulation in rice grains grown on Cd-contaminated soil.

There are many transport processes involved in Cd accumulation in rice grains, including root uptake, root-to-shoot translocation by xylem, transfer from xylem to phloem and remobilization of Cd from leaves to grains [5–7]. Previous studies have identified several loci controlling these processes in rice plants by using quantitative trait locus (QTL) analyses. Three QTLs on chromosomes 3, 6, and 8 were identified as influencing Cd concentration in brown rice [8]. A major QTL *qlGCd3* for low grain Cd content was identified on chromosome 3 [9]. Two QTLs, *qGCd7* and *qCdT7,* for grain Cd accumulation and Cd translocation, respectively, were detected on chromosome 7 [10, 11]. Recently, several transporters involved in these processes have been characterized in rice plants. Natural resistance-associated macrophage protein (NRAMP) family proteins play an important role in entry of Cd into rice root cells [12, 13]. *OsNRAMP5* was mainly expressed in the plasma membrane of rice root and participated in manganese (Mn) and Cd uptake. *Osnramp5* mutant resulted in significantly reduced Mn and Cd concentrations in rice straw and grain [14]. *OsNRAMP1* showed transport activity for Fe and Cd uptake, and was more highly expressed in high Cd-accumulating rice than low Cd-accumulating one [15]. Zinc-regulated transporter/iron-regulated transporter-like protein (ZIP) proteins and pleiotropic drug resistance (PDR)-type ATP-binding cassette (ABC) transporters have been also reported to be involved in Cd uptake and efflux in plants. Iron (Fe) deficiency-inducible expressions of *OsIRT1* and *OsIRT2* enhanced Cd uptake and translocation in rice [16]. AtPDR8 conferred Cd tolerance in Arabidopsis by pumping Cd^2+^ out across the plasma membrane of root epidermal cells [17]. *OsABCG36/OsPDR9* expression was significantly induced in rice roots by Cd exposure [18]. In addition, P-type ATPase heavy metal transporters (HMA) have been identified as the regulator for xylem Cd transport in rice by mediating vacuolar sequestration of Cd in rice roots [19]. Over-expression of *OsHMA3* in rice plants enhanced Cd tolerance and reduced Cd accumulation in rice grain. These studies provide deep insight into the genetic basis of Cd uptake and translocation in rice. However, the comprehensive expressions of these genes in low Cd-accumulation rice and its relationship to Cd accumulation have been little investigated in rice, especially for *indica* rice genotypes.

In past decades, microarray analysis has been a powerful tool providing important information on plants response to heavy metal stresses at the transcriptomic level. The comprehensive transcriptomic changes have been reported by various microarray analyse in rice and barley in response to Cd, copper (Cu) and mercury (Hg) stress [20–22]. These microarrays analyses improved our understanding of heavy metal-responsive gene expression in plants. However, microarrays are only applicable for detecting known transcripts and always miss low abundance genes, leading to a loss of some important candidate gene information. Recently, high-throughput RNA sequencing (RNA-seq) approach has become increasingly popular in transcriptomics studies, with increased sequencing depth and high detection sensitivity. To date, this technique has been applied in identifying the genes related to heavy metal responses in rice plants. Liu et al. [23] and He et al. [24] detected arsenite-responsive miRNAs and Cd toxicity-responsive genes in rice roots using RNAseq-based approach, and found that miRNAs and post-transcriptional alternative splicing may be involved in rice response to As and Cd exposure, respectively. Tan et al. [18] revealed universal Cd-responsive genes in rice roots exposed to various Cd stresses. Differential long non-coding RNAs were detected at an early stage in the rice response to Cd stress, suggesting their role in response to Cd stress [25]. Although these studies have identified several genes and transcription factors involved in response to heavy metal stresses in rice, the molecular mechanism underlying Cd uptake, translocation and accumulation has not been fully understood in low Cd accumulation rice.

In this study, 9311 and its mutant with extremely low Cd content in rice grains were used to investigate the differences in Cd uptake and accumulation in rice seedlings exposed to Cd stress. The candidate gene of low Cd accumulation mutant was identified by MutMap method and the comprehensive transcriptional changes related to Cd accumulation were determined using RNA-seq. Moreover, the tissue specificity of the candidate gene and its temporal response to Cd exposure in *lcd1* and WT roots were examined by quantitative real time RT-PCR. The objective of this work is to clarify the physiological and molecular mechanism in response to Cd stress in low Cd accumulation rice, which will be helpful for developing low Cd accumulation rice by marker-assisted breeding.

## Methods

### Plant culture and treatment

Seeds of *indica* rice (cv. Yangdao 6, namely 9311), provided by the Jiangsu Lixiahe Agricultural Research Institute, were subjected to ethyl methanesulfonate (EMS) mutagenesis. Approximately 450 g seeds were immersed in distilled water for 12 h, and then incubated in 1% EMS for 12 h. After the EMS treatment, the seeds were washed in distilled water. The low Cd accumulation mutant (namely *lcd1*) in rice grains was identified according to the grain Cd concentration of 35,689 M2 plants determined by inductively coupled plasma-mass spectrometry (ICP-MS; X Series 2, Thermo Fisher Corp., Waltham, MA, USA), and was further self-pollinated to obtain M5 generation of *lcd1*. The mapping population was generated from the cross between the M5 generation of *lcd1* and WT and was grown in Yoshida’s nutrient solution containing 0.1 μM Cd [26]. The F2 individuals with extremely high and low Cd accumulation in rice leaves were isolated at seedling stage and used as the mapping population for the MutMap method.

Field experiments were conducted during rice growing season from 2016 to 2017. Seeds of *lcd1* and WT were sterilized in 10% NaClO for 20 min and washed with deionized water. Then seeds were germinated on moist filter paper at 37 °C and sown in sterilized moist quartz sand. Uniform 25-day-old seedlings were subsequently respectively transplanted into the Cd-contaminated experimental paddy field in Hangzhou (Field A, 1.5 mg kg^− 1^ of total Cd, pH 5.4), Quzhou (Field B, 2.6 mg kg^− 1^ of total Cd, pH 5.8), Yiyang (Field C, 4.5 mg kg^− 1^ of total Cd, pH 4.8) and Xiangtan (Field D, 0.35 mg kg^− 1^ of total Cd, pH 5.2). Rice plants grown in different experimental paddy fields were uniformly managed and harvested at tillering, heading, filling and maturity stages, respectively. All samples were oven dried to constant weight at 70 °C for mineral element concentration analysis.

Hydroponics experiments were also conducted to investigate the effects of Cd stress on growth and Cd accumulation of *lcd1* and WT rice seedlings. Sterilized rice seeds were germinated on moist filter paper at 37 °C and grown on a plastic mesh floating on half-strength Yoshida’s nutrient solution. Uniform 15-day-old seedlings were transferred into fresh nutrient solutions for different Cd concentration treatments (0 and 5 μM). Each treatment was conducted in five replicated runs. After different times (0, 3, 6, 12 h and 14 days) of Cd treatment, rice plants were carefully washed with deionized water and separated into roots and shoots, immediately frozen in liquid nitrogen and then stored at − 80 °C until use.

### Kinetic analyses of cd uptake in roots

The Cd uptake in roots was determined according to Uraguchi et al. [27] with some modifications. Uniform 15-day-old seedlings of *lcd1* and WT were transferred to the uptake solution containing 0.5 mM CaCl_2_ and 2 mM MES (pH 5.6) for 24 h, and then carried out at room temperature for the uptake experiments.

For the dose-dependent Cd uptake experiment, rice seedlings were transferred to 1-L plastic container with the uptake solution containing different concentrations of CdCl_2_ (0, 0.1, 0.25, 0.5, 1, 5, 10, 15, 30 and 50 μM). Each treatment was replicated three times. After 1 h of uptake, plants were carefully washed with deionized water and separated into roots and shoots, oven dried and digested with HNO_3_ for Cd concentration analysis. Values of *Km* and *Vmax* for each genotype were calculated using software (GraphPadPRISM4; GraphPad Software Inc., CA, USA).

For the time-course Cd uptake experiment, rice seedlings were transferred to 1-L plastic container with the uptake solution containing 5 μM CdCl_2_ for different time treatments (0, 5, 10, 30, 60, 90, 120 and 180 min). Each treatment was replicated three times. The concentration of Cd in roots was determined as described below.

### Determination of metal elements concentrations

The metal elements (Cd, Fe, Mn, Zn and Cu) concentrations were determined by ICP-MS according to Cao et al. [28]. Briefly, dried rice root, shoot and grain samples (0.25 g) were digested in concentrated HNO_3_, and diluted to 50 mL by deionized water for metal elements determination.

### MutMap analysis

DNAs for MutMap analysis were extracted from rice leaves with DNeasy Plant Mini Kit (Qiagen, Hilden, Germany). Two parents and two bulked DNA pools were used for MutMap analysis. The bulked DNA were prepared by mixing DNA equally from 31 F2 individuals with extremely high and low Cd accumulation in rice leaves respectively. DNA quality and concentration were checked using the NanoPhotometer® spectrophotometer (IMPLEN, CA, USA) and Qubit® DNA Assay Kit in Qubit® 2.0 Flurometer (Life Technologies, CA, USA). Sequencing libraries were generated using Truseq Nano DNA HT Sample preparation Kit (Illumina, USA) and were sequenced by Illumina HiSeq4000 platform (Illumina, CA, USA).

The raw data (WT parent: SRR8695240, *lcd1* parent: SRR8695241, extremely high Cd accumulation pool: SRR8695238, extremely low Cd accumulation pool: SRR8695239) were filtered by phred quality score to remove adapter sequences and low-quality bases. The clean reads were aligned to the 9311 reference genome (ftp://ftp.ensemblgenomes.org/pub/plants/release-36/fasta/oryza_indica/dna/) using BWA software. Alignment files were converted to BAM files through SAMtools software and applied to GATK to identify reliable SNPs. The SNP-index of two pools were calculated using sliding window methods and the difference between the SNP-index of two pools was calculated as ΔSNP-index.

### RNA-seq analysis

Total RNAs for RNA-Seq analysis were extracted from rice roots of *lcd1* and WT under Cd exposure with RNeasy Plant kit (Qiagen, Hilden, Germany). RNA quality and concentration were determined by NanoPhotometer® spectrophotometer (IMPLEN, CA, USA) and Qubit® RNA Assay Kit in Qubit® 2.0 Flurometer (Life Technologies, CA, USA). Sequencing libraries were generated using NEBNext® Ultra™ RNA Library Prep Kit for Illumina® (NEB, USA) and were sequenced on an Illumina Hiseq platform (Illumina, CA, USA).

The raw sequences for WT (SRR8718745, SRR8718748 and SRR8718749, three biological replicates) and *lcd1* (SRR8718744, SRR8718746 and SRR8718747, three biological replicates) libraries were filtered using phred quality score to remove adapter sequences and low-quality bases. In high-quality reads, the sequences were trimmed and mapped to the 9311 reference genome using Hisat2. The best match was used to annotate genes and assign gene ontology information. FPKM values (fragments per kilobase of exon model per million mapped reads) were calculated to estimate gene expression levels. False positive and false negative errors were corrected by calculating the FDR (false discovery rate) adjusted q-values. The DEGs (differentially expressed genes) between *lcd1* and WT under Cd exposure were selected based on normalized read count, and fold change (*lcd1*/WT) ≥ 1.5 and q-value < 0.05 were used as threshold values by using the DESeq R package (1.18.0). Gene ontology (GO) enrichment analysis of DEGs between *lcd1* and WT under Cd exposure was performed using the GOseq R-package with q-value < 0.05.

### Quantitative real-time RT-PCR

The tissue specificity of the candidate gene and its temporal expression under Cd exposure were examined by quantitative real time RT-PCR (qPCR). The mixture of first-strand cDNA from 1 μg of total RNA served as the templates for qPCR analysis using ReverTra Ace® qPCR RT Master Mix (Toyobo, Japan). The primers used for qPCR were designed by Primer Premier 5.0 (Premier, Palo Alto, California, USA) as listed in Additional file 1: Table S1. The qPCR reactions were run on a QuantStudio® 3 (Applied Biosystems, Thermo Fisher Scientific, Waltham, MA, USA), with a total reaction volume of 25 μL containing 2 μL of diluted cDNA template, 12.5 μL of SYBR Green Realtime PCR Master Mix, 1 μL of 10 μM forward and reverse primer, and 8.5 μL of nuclease-free water. The following reaction conditions were applied: incubation at 95 °C for 60 s, 40 cycles of denaturation at 95 °C for 15 s, annealing at 55 °C for 15 s and extension at 72 °C for 45 s, followed by the dissociation stage for melt curve analysis (from 60 to 94 °C). All of the samples were run with three replicates, and three no-template controls (NTC) were included in every run to monitor possible DNA contamination. *ACTIN-1* was used as a control to calculate the relative gene expression levels.

To optimize qPCR conditions for each primer pair, PCR amplification specificity and efficiency were examined according to the MIQE guidelines [29]. The specificity of the PCR amplification was checked with a melt curve analysis. The result showed that the amplified product yielded a single peak at the melting temperature (Tm) for each gene, indicating that primer pairs for qPCR are highly specific. The PCR amplification efficiency was assessed using 5-fold serial dilution calibration-curve. The slopes of calibration curves range from − 3.53 to − 3.48, with corresponding *R*^2^ and efficiencies of 0.988–0.997 and 92.1–93.9%, respectively (Additional file 2: Table S2). conforming that the PCR amplification efficiency for each primer pair meets the MIQE recommended range (90.0–105.0%). The samples were normalized using *ACTIN-1* expression and the relative gene expression levels were analyzed using the 2^(−ΔΔCT)^ method due to that the amplification efficiencies of the target and reference were similar (Additional file 2: Table S2).

### Statistics

Data were expressed as mean ± standard error of at least three biological replicates for each sample. Statistical analyses were performed using the statistical software graphpad prism (Graphpad Software, San Diego, CA, USA). Statistical significance was assessed using Duncan’s multiple comparison at *P* < 0.05 level.

## Results

### Isolation and characterization of the *lcd1* mutant

**Fig. 1:**
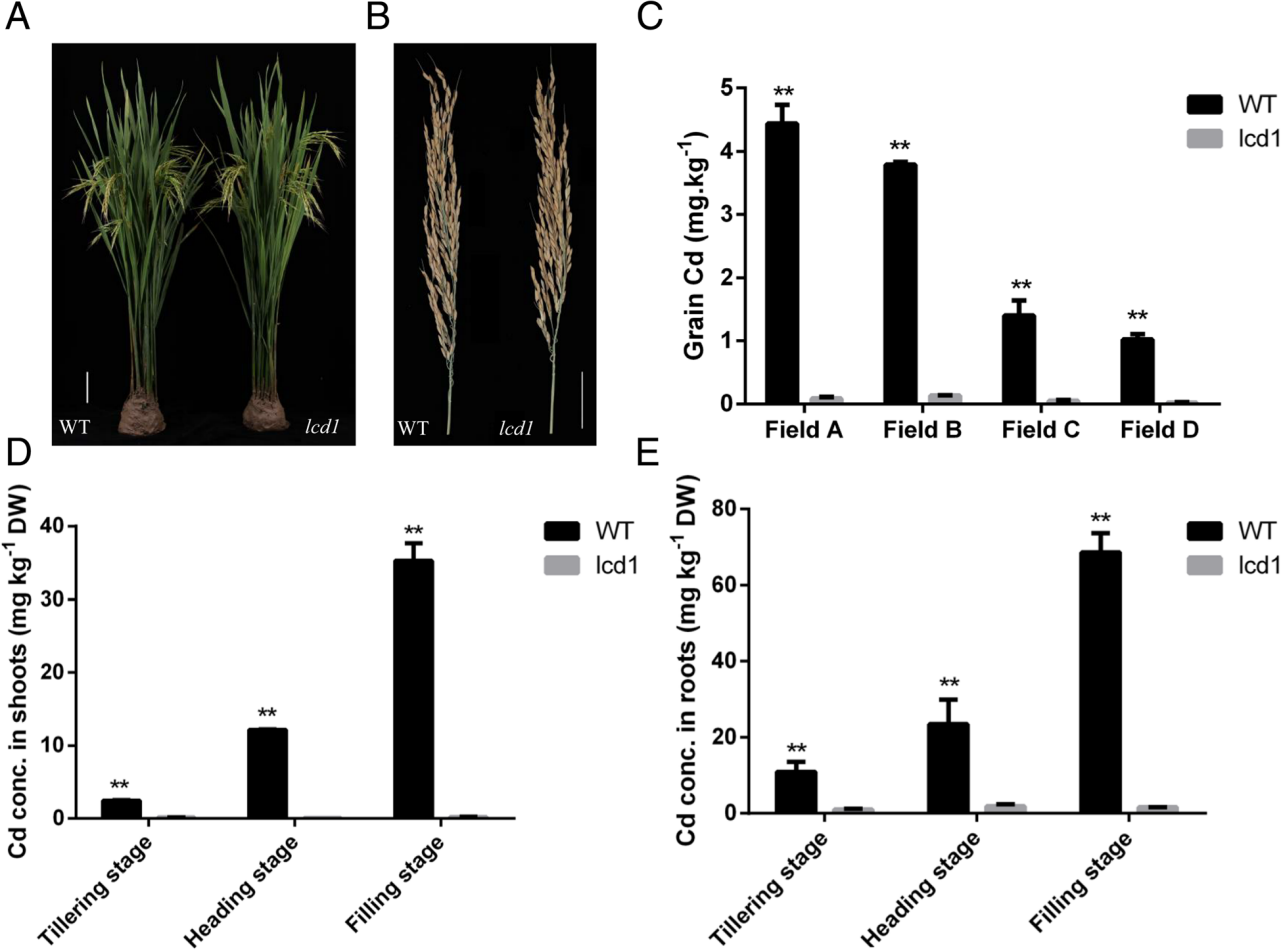
Plant morphology and Cd concentrations in *lcd1* and WT (cv.9311) plants and grains. **a**, *lcd1* and WT plants after heading, bar = 10 cm. **b**, *lcd1* and WT panicles, bar = 5 cm. **c**, Cd concentrations in *lcd1* and WT grains grown on Cd-contaminated fields. **d** and **e**, Cd concentrations in shoots and roots of *lcd1* and WT at tillering, heading and filling stage. * and ** indicate significant difference at *p* < 0.05 and *p* < 0.01 level between *lcd1* and WT, respectively. The same as below

We isolated a mutant (*lcd1*) displaying extremely low Cd content in rice grain from an EMS-mutagenized *indica* rice 9311. The *lcd1* mutant was identified in screening for grains Cd concentration from among 35,689 M2 plants grown on Cd-contaminated paddy field and was further self-pollinated to obtain M_5_ generation of *lcd1*. At maturity, there were no obvious differences in plant height, tiller number and panicle morphology between *lcd1* and WT grown in different paddy fields (Fig. 1a, b, Additional file 3: Table S3). However, the concentrations of Cd in *lcd1* grains from all paddy fields were extremely low, ranging from 0.02 to 0.13 mg·kg^− 1^, which was much lower than the maximum limit of 0.2 mg·kg^− 1^ for Cd in rice; whereas the corresponding Cd concentrations in WT grains were 1.02–4.44 mg·kg^− 1^ (Fig. 1c). The grain Cd concentrations in *lcd1* decreased by 96.2–98.0% compared with those in WT. Similar results were observed for root and shoot Cd concentrations of *lcd1* at tillering, heading and filling stages (Fig. 1d, e). The other metal concentrations, such as Zn, Cu and Fe in grains were not significantly different between *lcd1* and WT grown in most paddy fields. However, (Mn concentrations in *lcd1* grains from all paddy fields were significantly lower than those in WT (Additional file 4: Figure S1).

**Fig. 2:**
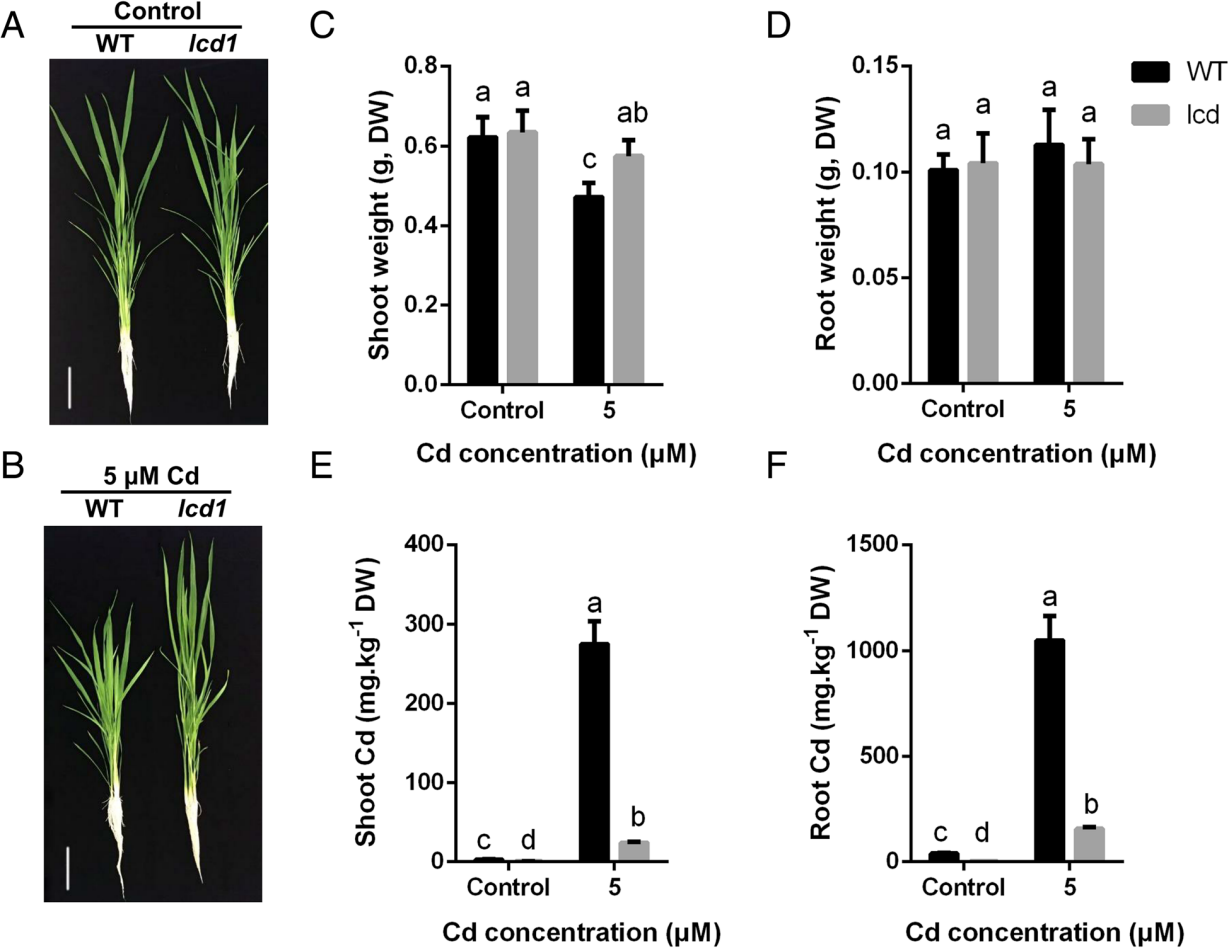
Plant biomass and Cd concentrations in *lcd1* and WT seedlings under Cd exposure. **a**-**d**, plant phenotype and biomass of *lcd1* and WT seedling exposed to 5 μM CdCl_2._ E and F, Cd concentrations of the shoots and roots in *lcd1* and WT seedling exposed to 5 μM CdCl_2._ Letters a-d indicate difference is significant at 0.05 level

Rice seedlings of *lcd1* and WT were exposed to 5 μM Cd in hydroponics. Under control condition, there were no visible differences in plant height, root length, root and shoot weights between *lcd1* and WT. When grown in the presence of 5 μM Cd, both *lcd1* and WT showed decreases in plant height and shoot weight compared with control, however, *lcd1* shoots were much longer than WT in the presence of 5 μM Cd (Fig. 2a-d). Moreover, *lcd1* accumulated less Cd in both shoots and roots compared with WT, irrespective of Cd treatment (Fig. 2e, f). The concentrations of Cu and Zn in both shoots and roots did not differ significantly between *lcd1* and WT, however, the concentrations of Mn in both shoots and roots were significantly lower in *lcd1* than in WT, while the shoot Fe concentration was relatively lower in *lcd1* than in WT (Additional file 5: Figure S2).

**Fig. 3:**
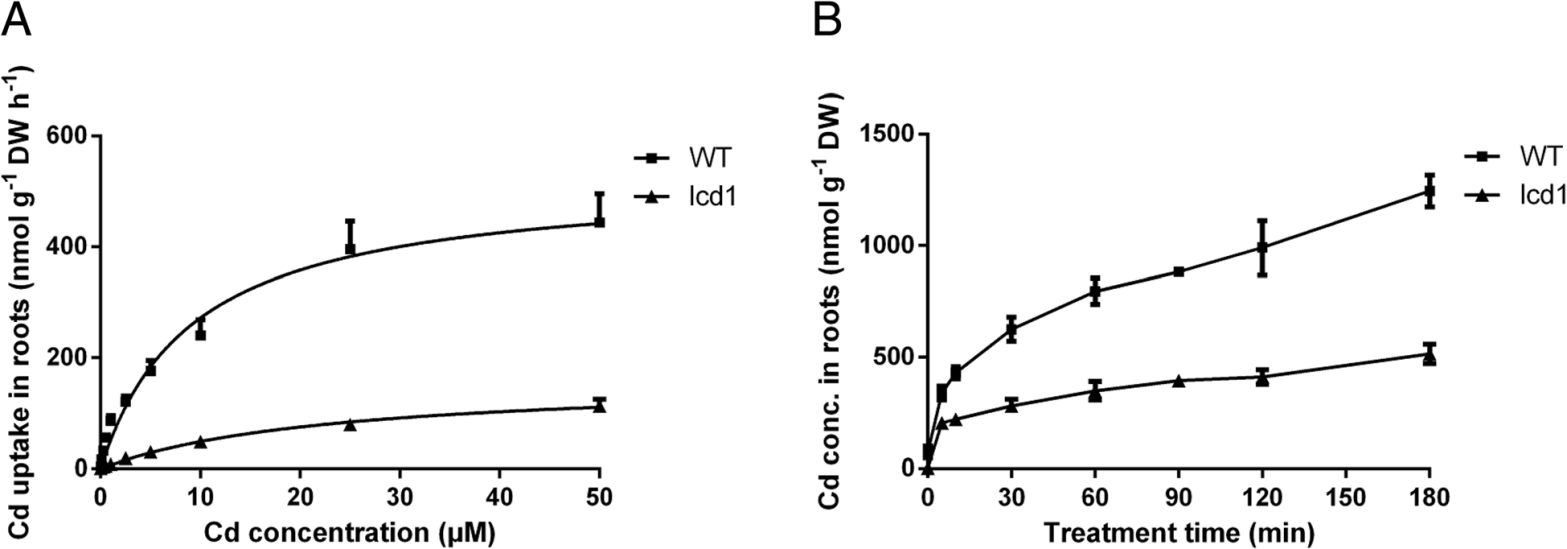
The dose- (**a**) and time-dependent (**b**) Cd uptake in roots of *lcd1* and WT seedlings

The Cd uptake abilities of *lcd1* and WT were further determined by a dose- and time-dependent Cd uptake in roots. The Cd uptake by roots was lower in *lcd1* than in WT under all Cd treatment concentrations (Fig. 3a). The *V*_*max*_ of *lcd1* (162.00 ± 9.11 nmol g^− 1^ DW h^− 1^) was lower than that of WT (523.70 ± 33.27 nmol g^− 1^ DW h^− 1^), and the value of *Km* was greater in *lcd1* (23.20 ± 2.83 μM) than in WT (9.26 ± 1.67 μM) (Table 1). The time-course of Cd uptake was 1.7–2.4 times higher in WT than in *lcd1* through 180 min of Cd exposure (Fig. 3b). These results show that the Cd uptake ability of *lcd1* is much lower than that of WT.

**Table 1:** The Michaelis-Menten equation parameters of root Cd uptake in *lcd1* and WT under different Cd exposure. Data are presented as means ± SE (*n* = 3)

Rice genotypes	*V*_*max*_ (nmol g^− 1^ DW h^− 1^)	*Km* (μM)	*r* ^*2*^
WT	523.70 ± 33.27	9.26 ± 1.67	0.9823
*lcd*	162.00 ± 9.11	23.20 ± 2.83	0.9950

### Genetic analysis and identification of the candidate region of the *lcd1* mutant by MutMap analysis

For rapid identification of the gene controlling the low Cd accumulation phenotype of the *lcd1* mutant, we crossed the *lcd1* mutant to WT 9311 in 2016 to obtain F1 progeny. F1 plants were self-pollinated to generate F2 progeny in 2017. 439 F2 individuals were grown in Yoshida’s nutrient solution containing 0.1 μM Cd. The progeny segregated in a 3:1 ratio (327:112) for WT phenotype and low Cd accumulation phenotype, respectively, indicating that the low Cd accumulation phenotype of the *lcd1* mutant is caused by a single recessive mutation. We combined DNA form 31 F2 progeny that displayed extremely high (namely H2_H31 pool) and low Cd accumulation phenotype (namely H2_L31 pool) and subjected them to whole-genome sequencing using Illumina HiSeq4000 platform. We obtained 22,199,781,900 and 20,232,345,600 cleaned bases for Pool H2_H31 and H2_L31, respectively. These reads were aligned to the 9311 reference genome using the BWA software resulting in the identification of 1055 SNP positions.

**Fig. 4:**
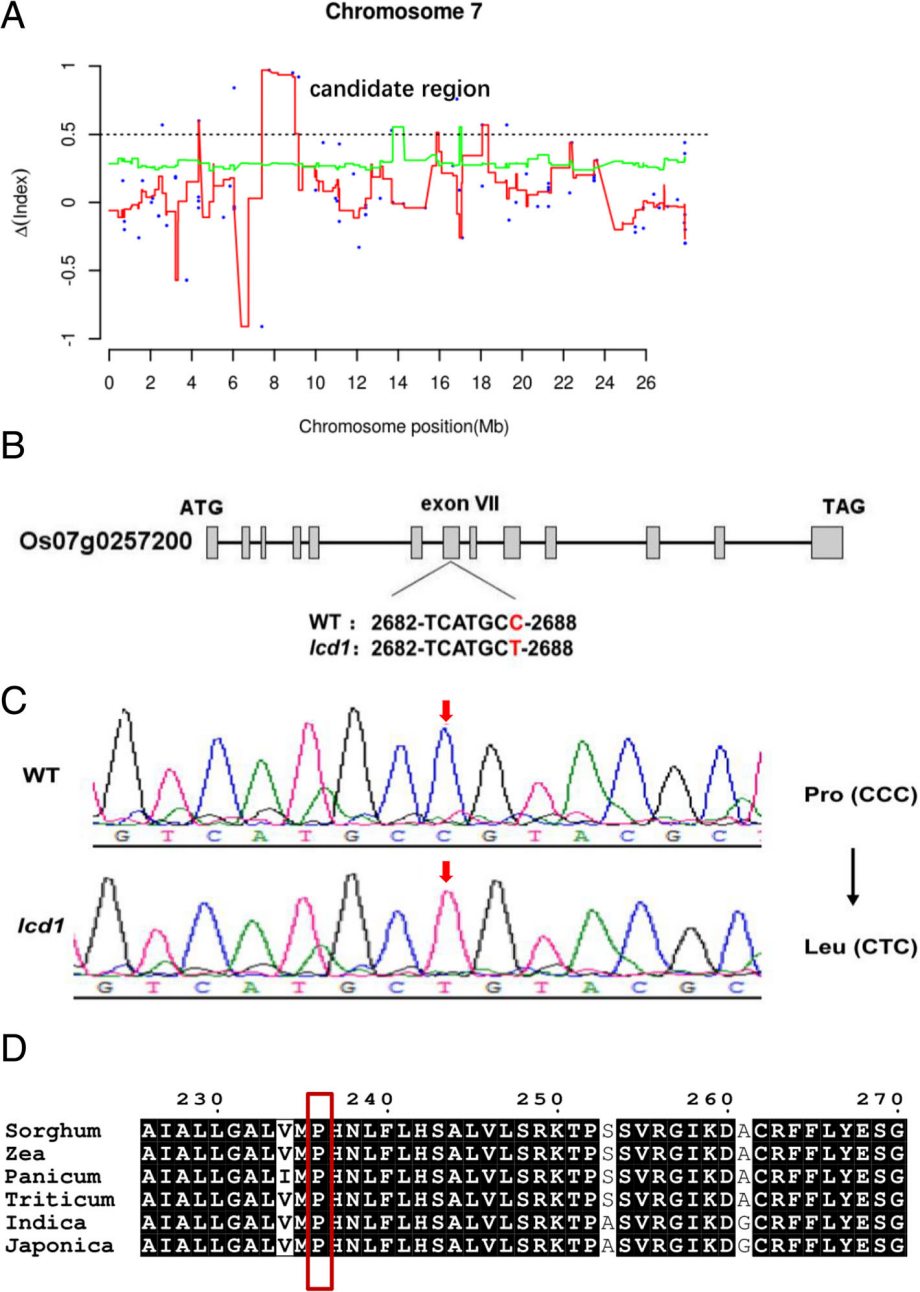
Identification of the *lcd1* mutant by MutMap. **a**, Δ SNP-index plots of two pools on chromosome 7 generated by MutMap analysis. **b**, Structure of the *OsNRAMP5* gene in *lcd1* and WT*.* Gray boxes and black lines represent exons and introns in the *OsNRAMP5* gene, respectively. SNP location is indicated below the gene structure with red font. **c**, Confirmation of SNP in the *lcd1* mutant by Sanger sequencing. The SNP is indicated with red arrow. **d**, Homology comparison of the OsNRAMP5 protein in different crop plants. * indicates the identical amino acids, red box indicates the amino acid mutation location

For each SNP position, the value of SNP-index was obtained and a graph relating SNP positions and ΔSNP-index (the difference of the SNP-index of two pools) were generated for all 12 chromosomes of rice. As we expected, ΔSNP-index was distributed randomly around 0 for most parts of the genome (Additional file 6: Figure S3). Finally, we obtained the candidate region containing a cluster of SNPs with ΔSNP-index values ranging from 0.6 to 1 on chromosome 7 (Fig. 4a).

### Identification of the causal SNPs of the *lcd1* mutant

There were nine SNPs with ΔSNP-index ranging from 0.6 to 1 identified in the candidate region, of which most SNPs located in non-coding region or had no gene annotation information (Table 2). A SNP at nucleotide position 8,887,787 corresponded to the seventh exon of the gene *OsNRAMP5* (BGIOSGA024510, Os07g0257200), previously identified as an important transporter responsible for Mn and Cd uptake. This SNP led to a C to T transition at codon 236, resulting in replacement of a Pro (CCC) residue by Leu (CTC) in OsNRAMP5 (Fig. 4b). Sequence analysis confirmed the mutation site in the *lcd1* mutant and multiple protein sequence comparison indicated that the residue affected was highly conserved in different crop plants (Fig. 4c, d), suggesting that the Pro236Leu substitution is likely responsible for the low Cd accumulation phenotype of the *lcd1* mutant.

**Table 2:** Summary of SNPs showing ΔSNP-index from 0.6 to 1 within the candidate region

ΔSNP-index	Position	Ref	Alt	Mutated gene	Mutation Type	Gene Annotation
0.95	8,887,787	C	T	BGIOSGA024510	Missense (P to L)	metal transporter Nramp5
0.92	9,174,237	C	T	BGIOSGA024502	Intron mutation	GHD7 protein
0.57	2,570,339	G	A	BGIOSGA024818	5′-UTR mutation	Hypothetical protein
0.57	18,066,507	C	A	BGIOSGA025813	3′-UTR mutation	Hypothetical protein
0.57	19,252,302	T	A	BGIOSGA025883	Missense (R to W)	Hypothetical protein
0.97	7,743,062	G	A	None	/	/
0.84	6,051,970	T	A	None	/	/
0.76	16,841,982	G	T	None	/	/
0.6	4,343,274	G	A	None	/	/

identified by MutMap analysis

Ref: reference base in WT, Alt: altered base in the *lcd1* mutant

**Fig. 5:**
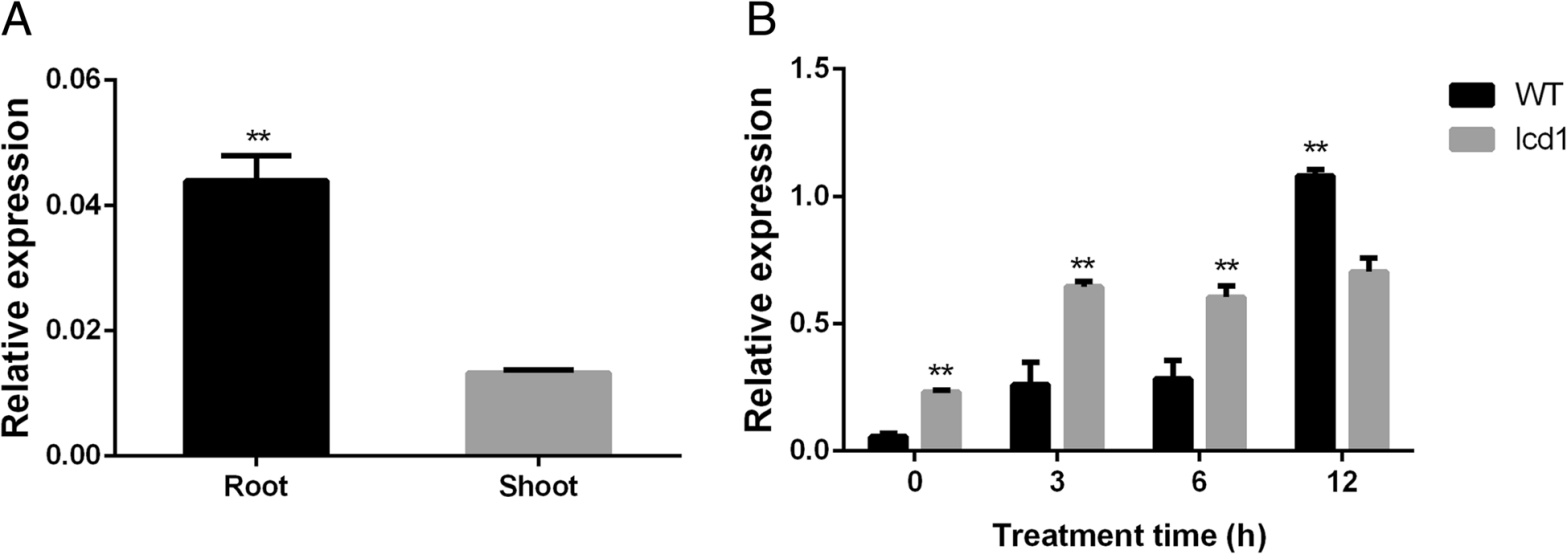
The tissue specificity of the *OsNRAMP5* gene and its temporal response to Cd exposure in *lcd1* and WT roots. **a**, Relative expression of *OsNRAMP5* in WT root and shoot. **b**, Temporal responses of *OsNRAMP5* in *lcd1* and WT roots to Cd exposure

The tissue specificity and temporal expression level of *OsNRAMP5* were further detected in *lcd1* and WT under Cd exposure. *OsNRAMP5* was expressed exclusively in WT root and little of its mRNA was detected in WT shoot (Fig. 5a). Moreover, *OsNRAMP5* expression markedly increased with enhancing Cd treatment time in both WT and *lcd1* roots compared with the control. However, *lcd1* root showed a higher O*sNRAMP5* expression at 3 and 6 h after Cd treatment, but significant lower expression at 12 h after Cd treatment than WT root (Fig. 5b), indicating a difference in Cd response pattern between *lcd1* and WT root.

### Transcriptome analysis of the *lcd1* mutant under cd exposure by RNA-seq

**Fig. 6:**
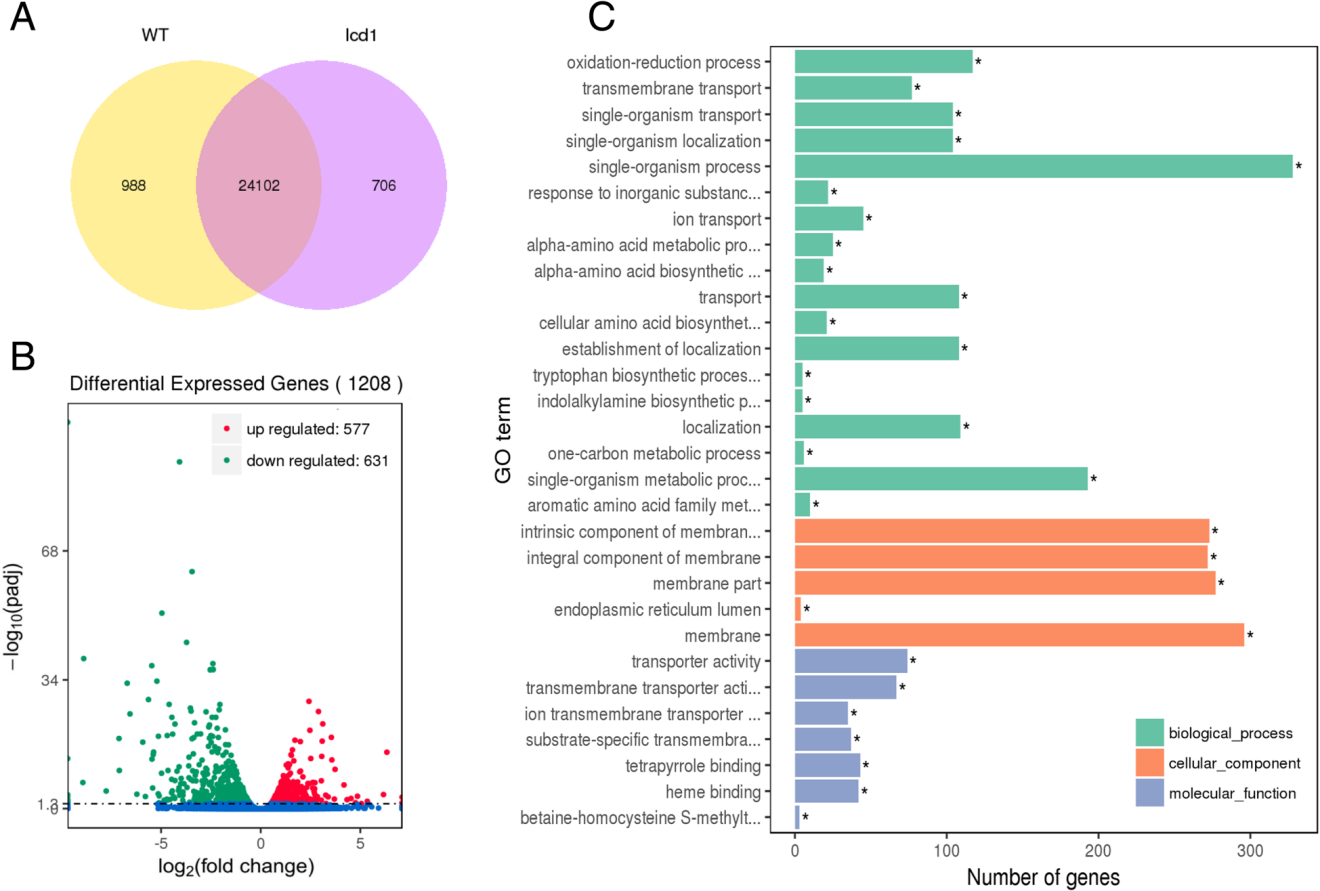
Transcriptional changes between *lcd1* and WT roots responsive to Cd exposure. **a**, Venn diagram of common Cd responsive genes in *lcd1* and WT. **b**, Volcano map of differentially expressed genes (DEGs) between *lcd1* and WT roots responsive to Cd exposure. **c**, Significantly enriched GO terms of DEGs between *lcd1* and WT roots responsive to Cd exposure. GO terms belong to biological processes, molecular functions, and cellular components were shown in green, red, and blue, respectively. GO terms were sorted based on q-values (*q*-value < 0.05)

To clarify how the mutation in *OsNRAMP5* affects transcript levels of other genes involved in Cd accumulation in rice roots. Transcriptome of *lcd1* and WT roots under Cd exposure were investigated by RNA-seq analysis. 15-day-old rice seedlings of *lcd1* and WT were exposed to 5 μM Cd for 6 h, and root samples were used for sequencing on an Illumina platform. An average of ∼51.5 million clean reads per sample was attained from *lcd1* and WT root cDNA libraries, among which more than 85% was uniquely mapped to the 9311 reference Genome (ftp://ftp.ensemblgenomes.org/pub/plants/release-36/fasta/oryza_indica/dna/). 25,090 expressed genes were detected in Cd-exposed WT root and 24,808 expressed genes in Cd-exposed *lcd1* root, among which 24,102 genes were common ones, indicating similar genetic background between the two genotypes (Fig. 6a). The list of detected genes from RNA-Seq for *lcd1* and WT under Cd exposure were provided in supplementary material (Additional file 7). A total of 1208 genes were differentially expressed between *lcd1* and WT roots using threshold values of fold change (*lcd1*/WT) ≥ 1.5 and q-value < 0.05, including 577 up-regulated and 631 down-regulated ones (Fig. 6b). GO classification of biological process, cellular component and molecular function of the DEGs were all significantly enriched in transmembrane transport (Fig. 6c), indicating that transmembrane transport process plays an important role in differential Cd accumulation between *lcd1* and WT roots.

The changes in transcriptional levels of NRAMP, ZIP, HMA and ABC family genes, previously identified as important genes involved in Cd transmembrane transport, were further analyzed between *lcd1* and WT (Table 3). In general, *lcd1* root showed relatively lower expressions in NRAMP and ABC family genes, and higher expressions in ZIP and HMA family genes than WT root under Cd exposure. Among them, four genes (*OsNRAMP1, OsABCG36/OsPDR9, OsZIP9* and *OsHMA3*) displayed larger relative changes in their expression levels between *lcd1* and WT roots under Cd exposure compared with the other genes, indicating that they are involved in differential Cd accumulation between *lcd1* and WT roots.

**Table 3:** Comparison of gene expressions related to Cd transmembrane transport between *lcd1* and WT roots under Cd exposure using RNA-seq analysis

Gene ID	Gene locus	Gene names	*lcd1* FPKM	WT FPKM	Fold change (*lcd1*/WT)
OsNRAMP family					
BGIOSGA025476	Os07g0258400	*OsNRAMP1*	6.20	19.44	0.33
BGIOSGA011239	Os03g0208500	*OsNRAMP2*	19.56	24.24	0.83
BGIOSGA020686	Os06g0676000	*OsNRAMP3*	40.09	62.85	0.66
BGIOSGA007187	Os02g0131800	*OsNRAMP4*	217.56	147.96	1.51
BGIOSGA024510	Os07g0257200	*OsNRAMP5*	47.21	43.86	1.11
BGIOSGA001567	Os01g0503400	*OsNRAMP6*	12.06	14.16	0.88
BGIOSGA035937	Os12g0581600	*OsNRAMP7*	9.67	12.98	0.77
OsZIP family					
BGIOSGA010092	Os03g0667500	*OsIRT1*	1.99	2.49	0.80
BGIOSGA010094	Os03g0667300	*OsIRT2*	0.02	0.02	1.06
BGIOSGA000021	Os01g0972200	*OsZIP1*	0.26	0.23	1.10
BGIOSGA010542	Os03g0411800	*OsZIP2*	70.14	93.40	0.75
BGIOSGA014364	Os04g0613000	*OsZIP3*	0.91	1.12	0.82
BGIOSGA028188	Os08g0207401	*OsZIP4*	44.19	27.47	1.61
BGIOSGA020032	Os05g0472700	*OsZIP5*	72.79	46.94	1.55
BGIOSGA018749	Os05g0164800	*OsZIP6*	58.52	60.36	0.97
BGIOSGA019328	Os05g0198400	*OsZIP7*	91.10	72.76	1.25
BGIOSGA025423	Os07g0232800	*OsZIP8*	18.26	12.97	1.41
BGIOSGA020030	Os05g0472400	*OsZIP9*	41.71	14.70	2.84
BGIOSGA023103	Os06g0566201	*OsZIP10*	22.26	9.91	2.25
BGIOSGA018313	Os05g0316100	*OsZIP11*	39.91	34.43	1.16
OsHMA family					
BGIOSGA020639	Os06g0690700	*OsHMA1*	12.91	11.42	1.13
BGIOSGA020589	Os06g0700700	*OsHMA2*	101.99	95.74	1.07
BGIOSGA024568	Os07g0232900	*OsHMA3*	12.45	9.80	1.27
BGIOSGA007732	Os02g0196600	*OsHMA4*	105.93	82.41	1.29
BGIOSGA016903	Os04g0556000	*OsHMA5*	42.69	37.76	1.13
BGIOSGA007052	Os02g0172600	*OsHMA6*	19.51	21.01	0.93
BGIOSGA028879	Os08g0486100	*OsHMA7*	20.66	17.04	1.21
BGIOSGA011343	Os03g0178100	*OsHMA8*	12.85	11.85	1.08
BGIOSGA020720	Os06g0665800	*OsHMA9*	74.27	68.57	1.08
OsABC transporter family					
BGIOSGA001233	Os01g0609300	*OsABCG36/OsPDR9*	0.34	2.70	0.13
BGIOSGA004262	Os01g0696600	*OsABCB4*	11.83	26.58	0.45
BGIOSGA028618	Os08g0398300	*OsABCA7*	3.15	7.53	0.42
BGIOSGA015573	Os04g0209200	*OsABCC14*	2.12	6.01	0.35

## Discussion

Rice is a major dietary intake source of Cd for Asian populations, therefore, reducing rice grain Cd accumulation is very important for human health [2, 4]. Selection and breeding of low Cd-accumulation rice is recognized as the most economical and effective approach to reduce rice grain Cd concentration and associated Cd intake risk for humans [30]. In this study, we succeeded to obtain a non-transgenic low Cd accumulating rice mutant, *lcd1,* by EMS mutagenesis from 9311, a well-known commercial *indica* rice cultivar with its full genome sequence available. The *lcd1* mutant had the similar agronomic traits to WT 9311 when grown on moderately or severely Cd-contaminated fields, but accumulated extremely low Cd in grains and plants (Additional file 3: Table S3, Fig. 1). Physiological studies in hydroponic culture further showed that *lcd1* shoots were much longer than those of WT in the presence of 5 μM Cd, and had less Cd accumulation in shoots and roots compared with WT (Fig. 2). Moreover, root Cd uptake ability of *lcd1* was much lower than that of WT (Fig. 3, Table 1). These results indicate that the *lcd1* mutant has lower Cd-accumulating ability and higher tolerance to Cd stress than WT. It is noteworthy that the *lcd1* mutant do not show any inhibition in rice growth compared with WT, although the concentrations of Mn were significantly lower in *lcd1* rice seedling and grain than those in WT (Additional file 4: Figure S1, Additional file 5: Figure S2), suggesting the potential use of the *lcd1* mutant in reducing intake risk of Cd from rice consumption without yield penalty.

For rapid identification of the gene controlling the low Cd accumulation phenotype of the *lcd1* mutant, MutMap, a method based on whole-genome sequencing of bulked DNA of F2 segregating population was used in the present study. Compared with conventional map-based cloning, MutMap method can rapidly identify mutant genes and QTLs by using F2 progeny derived from the cross of the mutant and its wild type [31, 32]. Previous studies have identified some genes using MutMap method in rice. *OsRR22*, responsible for the salinity-tolerance phenotype for the *hst1* mutant, has been identified by a MutMap method [31]. *WB1*, a regulator of endosperm development in rice, has been identified by a modified MutMap method [33]. In the present study, MutMap analysis, sequencing verification and homologous comparison results showed that a Pro236Leu amino acid substitution in a highly conserved region of OsNRAMP5 is likely responsible for the low Cd uptake and accumulation phenotype of the *lcd1* mutant (Fig. 4). In line with our study, Ishikawa et al. [13] isolated three low Cd accumulation mutants from ion-beam irradiated seeds of *japonica* rice cultivar Koshihikari, namely *lcd-kmt1*, *lcd-kmt2* and *lcd-kmt3*. Gene identification results showed that low Cd accumulation phenotype of three mutants were caused by 1-bp deletion, 433-bp insertion and *~* 277 kb deletion in *OsNRAMP5* respectively. In the present study, we isolated the *indica* rice *lcd1* mutant with the nonsynonymous mutation at codon 236 (CCC → CTC) in *OsNRAMP5*. The *lcd1* mutant had similar extremely low Cd accumulation phenotype to the reported *OsNRAMP5* mutants [13], but differed in the mutation site of the *OsNRAMP5* gene, indicating that it may be a new *OsNRAMP5* allelic mutant in rice. Furthermore, Tang et al. [34] and Sasaki et al. [14] reported that knockout of *OsNRAMP5* using the CRISPR/Cas9 system and T-DNA insertion produced low Cd-accumulating *indica* rice (cv Huazhan) and *japonica* rice (cv Zhonghua11), respectively, indicating that OsNRAMP5 is a major transporter responsible for Cd uptake in rice. In the present study, a Pro236Leu amino acid substitution likely results in a defective OsNRAMP5 in the *lcd1* mutant. Our results supported previous findings that OsNRAMP5 is a major transporter responsible for Cd uptake in rice. However, in the present study, the *OsNRAMP5* expressions in *lcd1* root were increased at 3 and 6 h after Cd treatment, but decreased at 12 h after Cd treatment than those in WT root (Fig. 5b). This might be due to the fact that low Cd concentration in the *lcd1* mutant promotes Cd absorption by inducing *OsNRAMP5* expression during the early stage of Cd exposure.

To clarify the molecular mechanism underlying Cd uptake, translocation and accumulation in the *lcd1* mutant, transcriptome changes between *lcd1* and WT under Cd exposure were investigated by RNA-seq analysis. RNA-seq provides an effective approach to identify the whole transcriptome changes in rice responsive to heavy metal stresses. Previous studies have demonstrated the transcriptional changes in rice plants responsive to Cd using high-throughput RNA sequencing [18, 24, 25]. Similar transcriptome investigations were performed in maize [35], barley [36] and bean (*Phaseolus vulgaris* L.) [37] under Cd, Zn and Cu stress. In this study, a total of 1208 DEGs were identified between *lcd1* and WT roots under Cd exposure, and most were significantly enriched in transmembrane transport (Fig. 6), indicating that the transmembrane transport process contributes to differential Cd response and accumulation between *lcd1* and WT roots. Hence, the changes in transcriptional levels of NRAMP, ZIP, HMA and ABC family genes, previously identified as important genes involved in Cd transmembrane transport, were further examined between *lcd1* and WT (Table 3). Four genes, *OsNRAMP1*, *OsABCG36/OsPDR9*, *OsZIP9* and *OsHMA3* displayed larger relative changes in their expression levels between *lcd1* and WT roots under Cd exposure compared with the other genes. OsNRAMP1 was involved in uptake and accumulation of Fe and Cd in rice. Over-expression of *OsNRAMP1* in rice enhanced Cd accumulation in the leaves [15]. In this study, *OsNRAMP1* expression was markedly decreased by 3.1-fold in *lcd1* root compared with WT root at 6 h after Cd exposure, indicating that the low Cd uptake and accumulation in *lcd1* root could be partly explained by the decreased expression of *OsNRAMP1*. Moreover, OsHMA3 has been identified as major regulator for xylem Cd transport in rice. *OsHMA3* participates in the sequestration of Cd into vacuoles and affects root-to-shoot Cd translocation in rice. Overexpression of *OsHMA3* enhanced Cd tolerance in rice [19], while loss-of-function allele of *OsHMA3* resulted in high Cd accumulation in rice shoots and grain in some *Indica* rice cultivars [38]. In this study, OsHMA3 protein coding sequence in *indica* rice 9311 was identical to the type V alleles of OsHMA3 as reported by Yan et al. [38], which was proved to be functional by heterologous expression in yeast. There was no SNP and InDel detected in *lcd1 OsHMA3* compared with WT, indicating no loss of function mutation of *OsHMA3* in the *lcd1* mutant. Furthermore, *OsHMA3* expression was increased by 1.3-fold in *lcd1* root compared with WT root at 6 h after Cd exposure, suggesting that increased *OsHMA3* expression probably adds to the effect of OsNRAMP5 mutation to account for the significant decreases in Cd accumulation in rice plant and grain of the *lcd1* mutant. In addition to *OsHMA3*, some PDR-type ABC transporters have been found to participate in Cd detoxification in plants. *AtPDR8* conferred Cd tolerance in Arabidopsis by pumping Cd^2+^ out across the plasma membrane of root epidermal cells [17]. In this study, the expressions of four ABC transporter encoding genes were all significantly down-regulated in *lcd1* root compared with WT root under Cd exposure, of which *OsABCG36/OsPDR9* showed a 7.9-fold decreased expression. This might be due to that the lower Cd uptake and intracellular Cd level in the root cell of *lcd1* caused weaker induction of *OsABCG36/OsPDR9* expression in *lcd1* root compared with in WT root*.* Actually, previous studies have demonstrated that *OsPDR9* was differentially induced by Cd stress. Moons [39] suggested that *OsPDR9* was rapidly induced by Cd stress in roots of rice seedlings. Tan et al. [18] also found that *OsABCG36/OsPDR9* expression was remarkably induced by Cd in rice roots, which is basically consistent with our results, indicating the potential role of ABC transporters in regulating Cd tolerance in rice plants.

## Additional files

Additional file 1: **Table S1.** The primer sequences for quantitative real-time RT-PCR (DOCX 19 kb)

Additional file 2: **Table S2.** The PCR amplification efficiency for each primer pair (DOCX 19 kb)

Additional file 3: **Table S3.** Comparison of the main agronomic traits between *lcd1* and WT plants (DOCX 15 kb)

Additional file 4: **Figure S1.** Metal concentrations in *lcd1* and WT grains grown on Cd-contaminated fields. (TIF 158 kb)

Additional file 5: **Figure S2.** Metal concentrations in shoots and roots of *lcd1* and WT seedlings exposed to 5 μM CdCl_2. (TIF 86 kb)_

Additional file 6: **Figure S3.** Δ SNP-index plots of two pools on all 12 chromosomes generated by MutMap analysis. (TIF 494 kb)

Additional file 7: The list of detected genes from RNA-Seq for *lcd1* and WT under Cd exposure. co_exp: genes with the common expression for *lcd1* and WT; specific_exp: genes specific for *lcd1* and WT. (XLS 3494 kb)

## Data Availability

All the data is contained within the manuscript.

## Abbreviations

ABC: ATP-binding cassette transporter
DEGs: differential expressed genes
EMS: ethyl methanesulfonate
GO: gene ontology
HMA: heavy-metal ATPase
NRAMP: natural resistance-associated macrophage protein
PC: phytochelatin
QTL: quantitative trait locus
RNA-seq: RNA sequencing
ZIP: zinc-regulated transporter/iron-regulated transporter-like protein
