# Supplementary material for: Gene identification and transcriptome analysis of low cadmium accumulation rice mutant (lcd1) in response to cadmium stress using MutMap and RNA-seq
Source: BMC Plant Biol. 2019 Jun 11;19:250. doi: 10.1186/s12870-019-1867-y (PMC6560816; doi:10.1186/s12870-019-1867-y)
Supplement: Supplementary file 1 — Table S1. The primer sequences for quantitative real-time RT-PCR (DOCX 19 kb) [file 12870_2019_1867_MOESM1_ESM.docx]

**Table S1** The primer sequences for quantitative real-time RT-PCR

| Gene name | Forward primer (5'–3') | Reverse primer (5'–3') |
| --- | --- | --- |
| *OsNRAMP5* | CAGCAGCAGTAAGAGCAAGATG | GTGCTCAGGAAGTACATGTTGAT |
| *OsActin-1* | CATCTATGAAGGATATGCTCTC | CCGTTGTGGTGAATGAGT |
