# Supplementary material for: Gene identification and transcriptome analysis of low cadmium accumulation rice mutant (lcd1) in response to cadmium stress using MutMap and RNA-seq
Source: BMC Plant Biol. 2019 Jun 11;19:250. doi: 10.1186/s12870-019-1867-y (PMC6560816; doi:10.1186/s12870-019-1867-y)
Supplement: Supplementary file 2 — Table S2. The PCR amplification efficiency for each primer pair (DOCX 19 kb) [file 12870_2019_1867_MOESM2_ESM.docx]

**Table S2** The PCR amplification efficiency for each primer pair

| Gene name | Slope | *R*^2^ | Efficiency(%) |
| --- | --- | --- | --- |
| *OsNRAMP5* | -3.48 | 0.997 | 93.9 |
| *OsActin-1* | -3.53 | 0.988 | 92.1 |
