# Supplementary material for: Gene identification and transcriptome analysis of low cadmium accumulation rice mutant (lcd1) in response to cadmium stress using MutMap and RNA-seq
Source: BMC Plant Biol. 2019 Jun 11;19:250. doi: 10.1186/s12870-019-1867-y (PMC6560816; doi:10.1186/s12870-019-1867-y)
Supplement: Supplementary file 3 — Table S3. Comparison of the main agronomic traits between lcd1 and WT plants (DOCX 15 kb) [file 12870_2019_1867_MOESM3_ESM.docx]

**Table S3** Comparison of the main agronomic traits between *lcd1* and WT plants

| Agronomic traits | WT | *lcd1* |
| --- | --- | --- |
| Plant height (cm) | 129.1±8.4 | 126.4±7.9 |
| Tiller number | 8.2±0.6 | 8.3±0.8 |
| Panicle length (cm) | 25.5±3.0 | 24.3±1.3 |
| Grain number per panicle | 201.3±11.2 | 197.2±12.6 |
| Seed setting (%) | 86.8±5.6 | 83.7±2.7 |
| 1000-grain weight (g) | 28.1±1.1 | 27.6±0.8 |
